# KAP Degradation by Calpain Is Associated with CK2 Phosphorylation and Provides a Novel Mechanism for Cyclosporine A-Induced Proximal Tubule Injury

**DOI:** 10.1371/journal.pone.0025746

**Published:** 2011-09-28

**Authors:** Olga Tornavaca, Eduard Sarró, Gloria Pascual, Beatriz Bardaji, M. Angeles Montero, M. Teresa Salcedo, Maria Plana, Joan López-Hellin, Emilio Itarte, Anna Meseguer

**Affiliations:** 1 Fisiopatologia Renal, Centre d'Investigacions en Bioquímica i Biologia Molecular (CIBBIM), Institut de Recerca Vall d'Hebron, Hospital Universitari Vall d'Hebron, Barcelona, Spain; 2 Unitat de Bioquímica de Biociències, Departament de Bioquímica i Biologia Molecular, Universitat Autònoma de Barcelona, Bellaterra, Spain; 3 Unitat de Bioquímica de Medicina, Departament de Bioquímica i Biologia Molecular, Universitat Autònoma de Barcelona, Bellaterra, Spain; 4 Department of Pathology, Vall d'Hebron University Hospital, Barcelona, Spain; INSERM, France

## Abstract

The use of cyclosporine A (CsA) is limited by its severe nephrotoxicity that includes reversible vasoconstrictor effects and proximal tubule cell injury, the latter associated whith chronic kidney disease progression. The mechanisms of CsA-induced tubular injury, mainly on the S3 segment, have not been completely elucidated. Kidney androgen-regulated protein (KAP) is exclusively expressed in kidney proximal tubule cells, interacts with the CsA-binding protein cyclophilin B and its expression diminishes in kidneys of CsA-treated mice. Since we reported that KAP protects against CsA toxicity in cultured proximal tubule cells, we hypothesized that low KAP levels found in kidneys of CsA-treated mice might correlate with proximal tubule cell injury. To test this hypothesis, we used KAP Tg mice developed in our laboratory and showed that these mice are more resistant to CsA-induced tubular injury than control littermates. Furthermore, we found that calpain, which was activated by CsA in cell cultures and kidney, is involved in KAP degradation and observed that phosphorylation of serine and threonine residues found in KAP PEST sequences by protein kinase CK2 enhances KAP degradation by calpain. Moreover, we also observed that CK2 inhibition protected against CsA-induced cytotoxicity. These findings point to a novel mechanism for CsA-induced kidney toxicity that might be useful in developing therapeutic strategies aimed at preventing tubular cell damage while maintaining the immunosuppressive effects of CsA.

## Introduction

Kidney androgen-regulated protein (KAP) is a highly specific, tightly regulated protein of kidney proximal tubule cells [1]. We studied KAP transcriptional regulation in mouse kidney and reported a fine-tuned regulation of its mRNA by thyroid and sexual steroid hormones, growth hormone (GH) and insulin-like growth factor 1 (IGF-1) in proximal tubule segments [1]–[8]. The absence of significant homologies with other proteins or with known structural domains has greatly reduced the experimental approaches to elucidate KAP function, which has remained elusive since first described in 1979 [9]. Previously, using specific antibodies raised against KAP-derived synthetic peptides, we identified an apparent 20kDa molecular-weight protein that paralleled KAP mRNA in terms of cell distribution and androgen regulation [10]. We also found that KAP interacts with the cyclosporine A (CsA) binding protein cyclophilin B (CypB) [10], and observed that KAP protein levels are lowered in kidneys of CsA-treated mice [10]. Moreover, KAP protected from CsA-induced toxicity when transfected to the proximal tubule-derived PCT3 cell line [10].

The great clinical benefits of CsA in the improvement of graft survival rates in organ transplantation are associated with significant undesirable nephrotoxic effects that include reversible vasoconstriction and proximal tubule cell injury, the latter associated with chronic kidney disease progression [11]–[13]. The mechanisms underlying CsA-induced toxicity in proximal tubule cells have not been completely elucidated. Morphologic evidence suggests that early sublethal tubular damage is confined to the S3 segment of the proximal tubule [14]. Since KAP is exclusively expressed in proximal tubules, we hypothesized that suboptimal KAP levels after CsA treatment could relate to homeostatic and/or metabolic alterations which, affecting proximal tubule cell function, could lead to cell injury and death.

The present report aimed to determine the putative protective effect of KAP *in vivo* using a KAP transgenic mouse model that overexpresses KAP in proximal tubule cells 15]. Moreover, we focused on elucidating the mechanisms that promote CsA-dependent KAP degradation, and hypothesized that post-translational mechanisms such as phosphorylation/dephosphorylation-related events could contribute to control of physiological KAP levels modulation of its degradation.

## Results

### KAP Tg mice are protected against CsA-induced tubular damage

**Figure 1:**
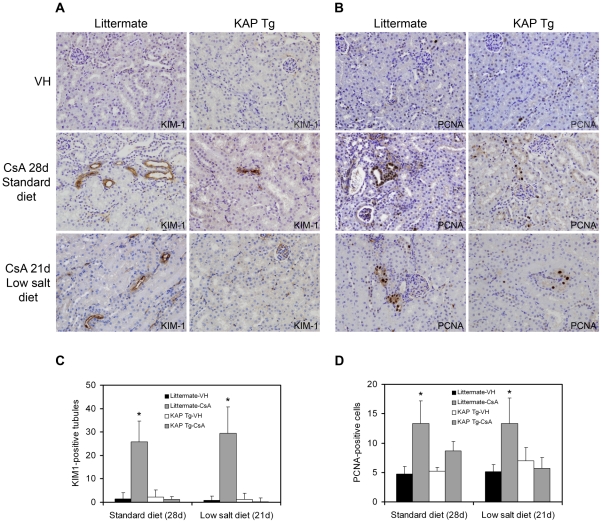
KAP Tg mice are protected from CsA-induced tubular damage. Expression levels of KIM-1 (A) and PCNA (B) were assessed by immunohistochemical staining in kidney sections of KAP Tg and control littermates treated with CsA, as indicated in Figure 1. Data shown are representative of six animals per group. Magnification: 400x. Quantitative analysis of KIM-1 positive tubules (C) and of PCNA-positive cells (D) of treatment conditions depicted in (A) and (B), respectively, are graphically represented. The number of PCNA-positive cells and KIM-1 positive tubules in each section was determined by counting positively stained cells or tubules in 10 randomly chosen fields (x 200 magnification) per slide. Data represent means ± SD of six animals per group. The littermate-CsA group has more KIM-1 positive tubules, for both standard (p<0.0001) and low-salt (p<0.0001) diets, compared to the littermate-VH, KAP Tg-VH and KAP Tg-CsA groups. Similarly, the littermate-CsA group has more PCNA positive tubules, for both standard (p<0.0001) and low-salt (p<0.0002) diets, compared to the littermate-VH, KAP Tg-VH and KAP Tg-CsA groups (ANOVA, with Tukey post-hoc test). *Significantly higher than KAP Tg-CsA.

We aimed to ascertain whether raised KAP levels in Tg mice would attenuate CsA-induced damage in proximal tubule cells. To this end, various doses of CsA were tested to select that which would produce tubular injury without clinical kidney damage in an attempt to observe the early effects of CsA on proximal tubule cells. Augmented SCr and BUN, together with interstitial tubular fibrosis, would reflect a clinical situation where early effects of CsA on tubular epithelia would be overdue. Early tubular injury can be assessed by expression of the kidney injury marker KIM-1 and the cell proliferation marker PCNA [16]–[18]. A 50 mg/kg/day dose of CsA was found to fulfill the exclusive tubular damage criteria when administered to animals fed either a standard diet for 28 days or a low salt diet for 21 days. SCr and BUN levels were unaffected under these CsA treatment conditions (Table S1), while KIM-1 and PCNA expression increased upon CsA administration in both standard and low-salt diet regimens in control littermates (Fig. 1A and 1B left panels). Quantitative results are represented in Figures 1C and 1D. KAP Tg mice exposed to the same treatments that caused tubular injury in littermates did not express KIM-1 (Fig. 1 right panels, and Fig. 1C) and no changes were observed in PCNA levels (Fig. 1B left panels, and Fig. 1D), thereby indicating that KAP protects proximal tubule cells from CsA-induced toxicity in vivo. Since KIM-1 and PCNA expression levels were unaffected by the type of diet in vehicle-treated mice (Fig. 1C and 1D), only a single representative picture for vehicle-treated control mice (VH) was included (Fig. 1A and 1B).

**Figure 2:**
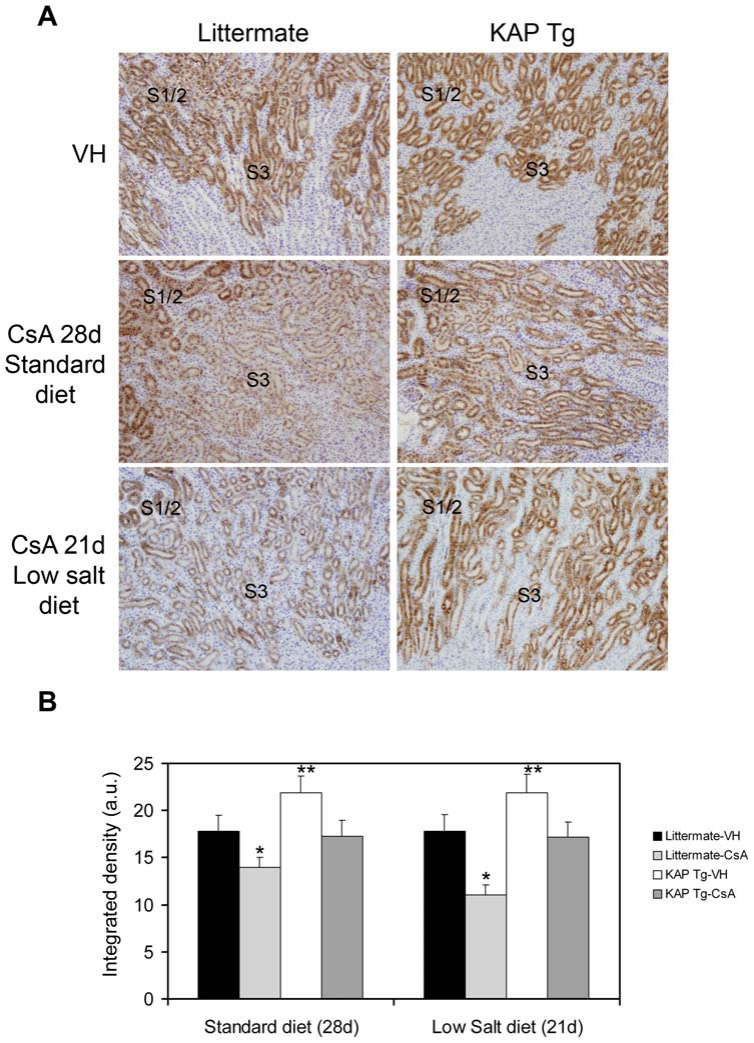
KAP levels in KAP Tg and control littermates after CsA treatment. (A) KAP expression levels were assessed by immunohistochemical staining in kidney sections of KAP Tg and control littermates using specific anti-KAP antibodies. Polyclonal antibodies against KAP were raised by rabbit immunization with the NH2-CPKIPLAGNPVSPTS-CONH2 KAP peptide. Mice were treated with CsA (50 mg/kg/day) for 28 days with standard diet or 21 days with a low-salt diet, as indicated in Fig 1. Vehicle-treated mice are marked as VH. Data shown are representative of six animals per group. Magnification: 40x. (B) Quantification of KAP levels in Tg and littermates, under control conditions and following different injuries, using the ImageJ software (ImageJ 1.44p, National Institutes of Health, Bethesda, Maryland, USA). Data represent means ± SD of six animals per group. There are statistical significant differences for both standard (p<0.0001) and low salt (p<0.0001) diets, being littermate-CsA KAP levels (*) lower than the others groups, and KAP Tg-VH KAP levels (**) higher than the other groups (ANOVA, with Tukey post-hoc test).

Results showed that in CsA-treated Tg mice, KAP levels in the S3 segment remained similar to those observed in vehicle-treated control littermates (Fig 2A and 2B). Quantification of the degree of KAP overexpression under control conditions and following different CsA treatments was made by using the ImageJ software, as indicated in Methods (Fig 2B). Since KIM-1 and PCNA levels were not increased in CsA-treated Tg mice (See Figure 1), we postulate that maintenance of KAP levels in proximal tubule cells prevents Tg mice from CsA-induced tubular damage.

### CsA activates calpain and caspases in mouse kidney and cultured proximal tubule cells

**Figure 3:**
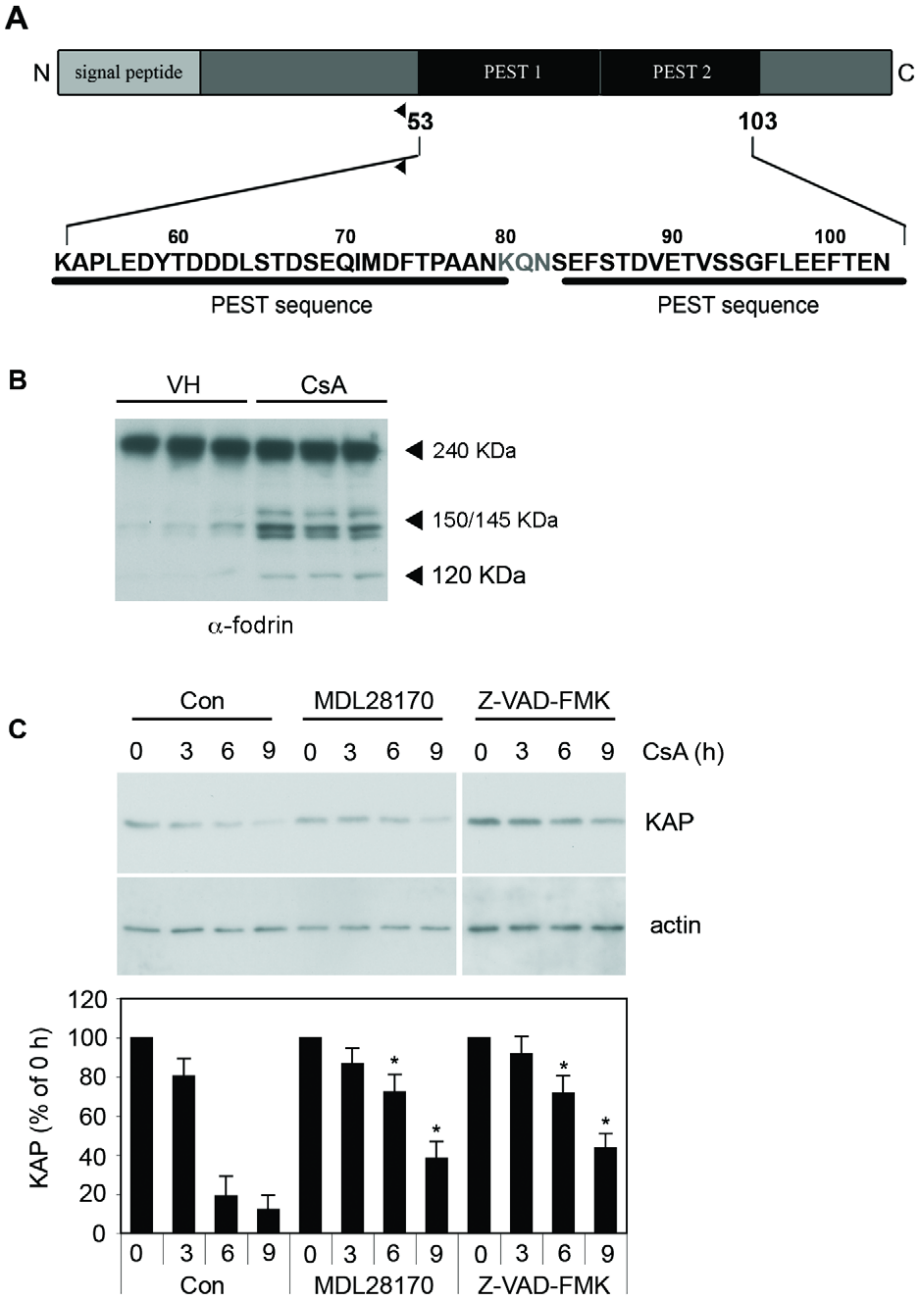
CsA activates calpain and caspases in kidney and cultured proximal tubule cells. (A) KAP protein analysis using the epestfind program (http://emboss.bioinformatics.nl/cgi-bin/emboss/epestfind) identified a new putative PEST sequence (PEST 1) between residues 53 and 89. This adds to the known putative PEST sequence (PEST 2) reported previously between residues 83 and 102. (B) Protease activation in mice treated with olive oil (vehicle) or CsA at 80 mg/kg/day for 14 days was analyzed by Western blot assay in crude kidney extracts. Degradation of α-fodrin (240-kDa) into the 150/145 kDa and 120 kDa fragments was determined to assess endogenous calpain and caspase activities, respectively. (C) KAP stability was analyzed in PCT3 transfected cells treated for 3, 6 and 9 h with CsA 10 µM alone or in combination with either calpain or caspase inhibitors (MDL28170 or Z-VAD-FMK, respectively). Actin levels were monitored as protein loading control (upper panel). Normalized densitometric values of the western blot signals are shown in the lower panel of Fig 3C. The amount of KAP at time 0 h was taken as the 100% in each case. Values at different times were referred to as a percentage of the total initial protein. *Significantly higher than the time-paired control (Student-t test, p<0,05).

Since KAP levels appeared to be critical in CsA-induced toxicity, we aimed to investigate the mechanisms involved in KAP degradation. Previous analysis of the KAP primary sequence reported a putative PEST sequence encompassing 83–102 residues 3]. More detailed analysis using the epestfind program (http://emboss.bioinformatics.nl/cgi-bin/emboss/epestfind) revealed a new PEST sequence between residues 53 and 89 with a PEST score of 6.35 (Fig. 3A). PEST sequences have been associated with proteins with a short half-life, and act as recognition signals for degradation via proteosome or calpain. Calpain activation was then analyzed by western blot assays in crude kidney extracts from CsA-treated mice using endogenous α-fodrin as a reporter. Results showed that, after CsA treatment, α-fodrin became cleaved in a pattern characteristic of calpain and caspases activation (150/145 kDa and 120 kDa fragments, respectively) (Fig. 3B) 19,20].

Since CsA activates calpain and caspases in mouse kidney and KAP is degraded in response to this immunosuppressant (Fig 2), we aimed to ascertain whether calpain and caspases contributed to CsA-induced KAP degradation. To this end, we used the PCT3 proximal tubule cell line [8], and determined the effects of the calpain inhibitor MDL28170 and the pan caspase inhibitor Z-vad-FMK on KAP stability. As shown in Fig 3C, KAP levels were significantly preserved in the presence of both inhibitors, thereby suggesting that CsA-induced KAP degradation might be mediated by calpain and caspases in this cell line.

### Protein kinase CK2 is involved in KAP phosphorylation in cultured cells

**Figure 4:**
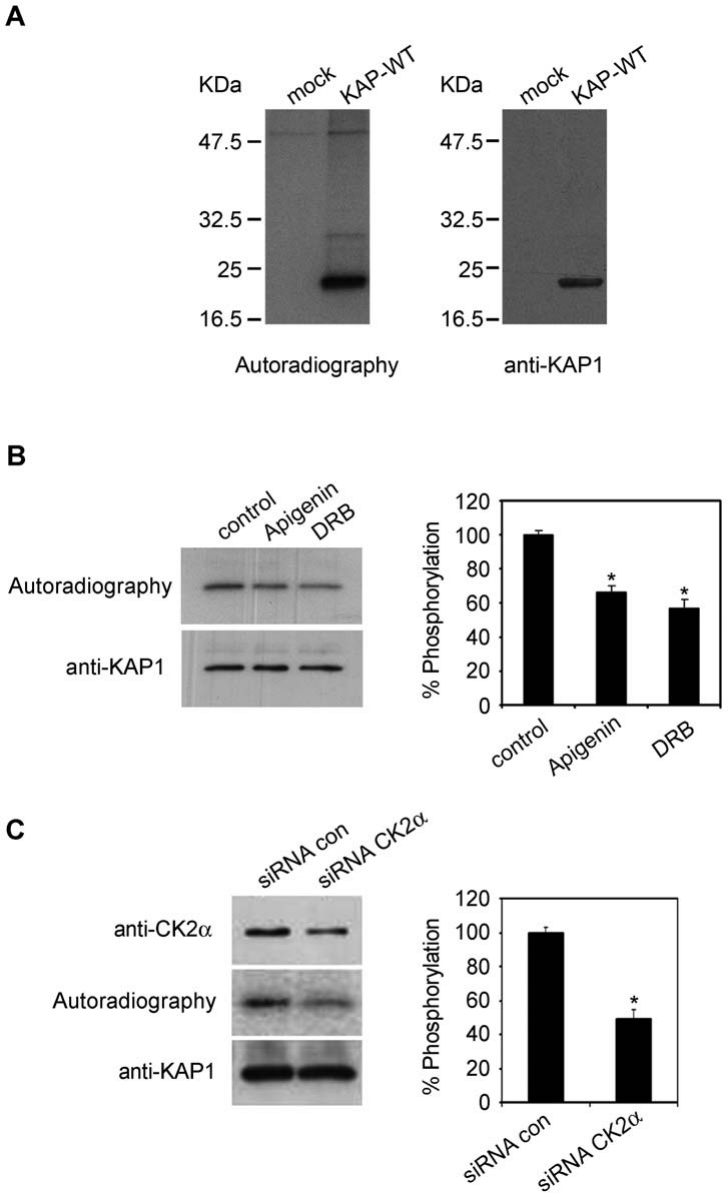
Protein kinase CK2 is involved in KAP phosphorylation in cultured cells. (A) Transient transfected PCT3 cells with an empty vector (mock) or containing the KAP-WT construct were metabolically labeled with [^32^P]orthophosphate. Cell lysates were immunoprecipitated with the anti-HA antibody and the products resolved in an SDS-PAGE, transferred to a PVDF membrane and exposed to an X-ray film (left panel). Western blot with specific anti-KAP-1 antibodies demonstrates that the phosphorylated protein was KAP (right panel). (B) KAP-WT transfected PCT3 cells were exposed to the CK2 inhibitors apigenin and DRB for one hour before exposure of the cells to [^32^P]orthophosphate and for the ensuing three hours of metabolic labeling. Densitometric autoradiographic values were referred to values of Western blot assays performed with an anti-KAP antibody (left panel). The percentage of phosphorylation inhibition obtained in the presence of apigenin and DRB, in relation to the control situation, is graphically represented (right panel). *Significantly lower than control (Student-t test, p<0,05). (C) PCT3 cells were co-transfected with KAP-WT and either non-silencing control (siRNA con) or anti-CK2α siRNA. After 48h of transfection, the cells were exposed to [^32^P]orthophosphate, labeled for 3h and processed as indicated in (A). *Significantly lower than siRNA control (Student-t test, p<0,05).

PEST sequences are frequently conditional signals, and the protein is not recognized until it is “marked” for degradation [21]. Phosphorylation is one of the molecular mechanisms described for activating this process [22]. In this respect, Prosite sequence analysis predicted Thr60, Thr66 and Thr87 within the PEST sequence as potential phosphorylation sites for protein kinase CK2, fulfilling the minimal consensus sequence for this kinase (Ser/Thr-X-X-Asp/Glu). Ser65 and Ser86 can also be considered as potential CK2 sites according to the requirements fulfilled by other CK2 phosphorylated proteins [23] (Fig. 3A). All these sites were also predicted to be CK2 phosphorylation sites by the NetPhosK 1.0 Server. These data suggest that CK2 phosphorylation of KAP could favor its degradation. Thus, we aimed to ascertain whether KAP was phosphorylated in transiently-transfected PCT3 cells metabolically labeled with [^32^P]orthophosphate. Labelled-immunoprecipitates showed radioactive phosphate incorporation into a protein (Fig. 4A, left panel) recognized by specific anti-KAP antibodies (Fig. 4A, right panel). Mock-transfected cells were negative, thereby demonstrating that KAP phosphorylation was specific.

To determine whether CK2 was the kinase responsible for KAP phosphorylation, KAP-WT transfected PCT3 cells were exposed to the CK2 inhibitors apigenin and DRB for one hour prior to exposure of the cells to [^32^P]orthophosphate and for the ensuing three hours of metabolic labelling. Both inhibitors reduced KAP-WT phosphorylation (Fig. 4B). In order to further demonstrate CK2 involvement in KAP phosphorylation, we carried out CK2α siRNA-based experiments using KAP-WT transfected PCT3 cells and showed that KAP phosphorylation decreased around one-half when CK2α expression was reduced to 50% (Fig. 4C).

**Figure 5:**
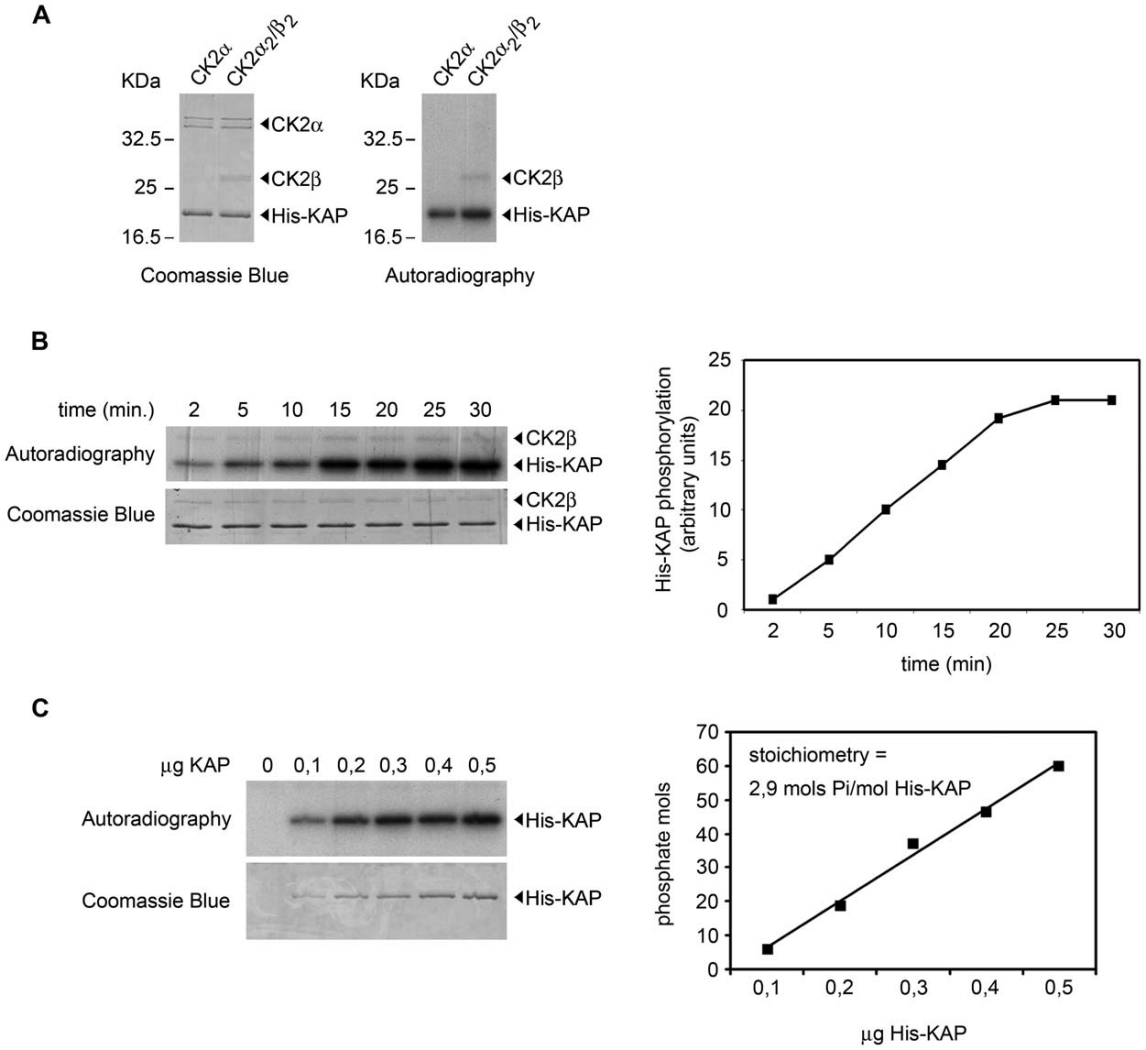
KAP is a substrate for protein kinase CK2 *in vitro*. (A) Ability of CK2 to phosphorylate KAP *in vitro* using mouse recombinant His-KAP-purified protein as substrate and recombinant purified CK2 as the kinase in the presence of [-^32^P]ATP (Amersham Biosciences). Products were resolved in a 15% SDS-PAGE and visualized with Coomassie Blue staining. Phosphorylated His-KAP recombinant protein was detected by autoradiography. (B) Time-course phosphorylation assay. 0.5 µg of His-KAP purified protein were incubated in the presence of 2 pmols of the holoenzyme. Products were resolved in a 15% SDS-PAGE and visualized with Coomassie Blue staining. Phosphorylated His-KAP recombinant protein was detected by autoradiography (left). Ratios between autoradiographic and Coomassie densitometric signals were graphically represented (right). (C) Stoichiometry of the His-KAP phosphorylation. Increasing amounts of mouse recombinant His-KAP-purified protein were incubated for 30 min in the presence of 2 pmols of the holoenzyme. CK2 was able to incorporate up to 2.9 mols of phosphate/mol of His-KAP, indicating that there are at least three sites that can be phosphorylated by the kinase.

The ability of CK2 to phosphorylate KAP was also demonstrated *in vitro* using recombinant His-KAP-purified protein and recombinant CK2α or the reconstituted holoenzyme CK2(α_2_/β_2_) (Fig. 5A and B). Stoichiometry analysis, with increasing quantities of His-KAP protein (0.1 to 0.5 µg) incubated with the holoenzyme to maximum phosphorylation (30 min), showed that CK2 incorporated up to 2.9 mols of phosphate/mol of His-KAP, therefore indicating the presence of at least three CK2 phosphorylation sites in KAP protein (Fig. 5C).

### KAP is phosphorylated at residues located in the PEST sequence

**Figure 6:**
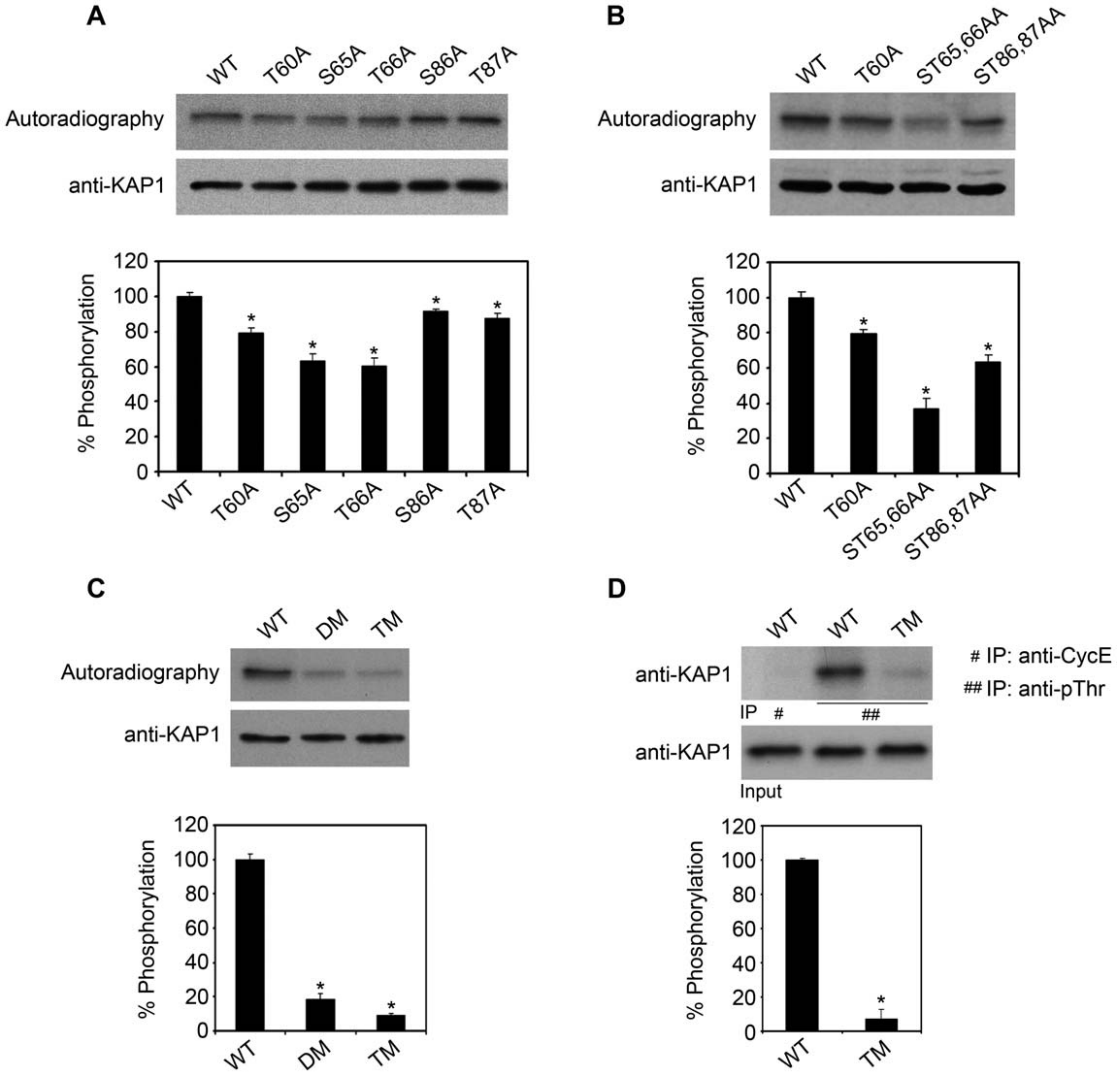
In cultured kidney cells KAP, is phosphorylated at residues located in the PEST sequence. (A) Residues Thr60, Ser65, Thr66, Ser86 and Thr87 were mutated and KAP phosphorylation analyzed in ^32^P-labeled PCT3 cells overexpressing KAP mutants. Numbers in the lower panel of Fig. A and in the following panels were calculated by densitometric analyses of phosphorylated protein and normalized by total KAP protein levels. The ratio corresponding to KAP-WT protein was used as a reference in each case. * p<0,05 vs WT (Student-t test). (B) Individual domains that include T60A, ST65/66AA and ST86/87AA were also tested. As depicted in Fig. B, all three constructs resulted in a reduction in KAP phosphorylation. * p<0,05 vs WT (Student-t test). (C) New mutants that include T60A and ST65/66AA (KAP-DM) and residues T60A, ST65/66AA and ST86/87AA (KAP-TM) were also produced. The phosphorylation capacities of DM and TM constructs were assessed as explained in part A of this figure. * p<0,0001 vs WT (Student-t test).(D) Immunoprecipitation of KAP-WT and KAP-TM in transfected PCT3 cell extracts using anti-phospho-Thr antibodies (##), and further immunoblotting with anti-KAP specific antibodies showed that the Thr residues mutated in the KAP-TM form are phosphorylated in the WT form. Immunoprecipitation of KAP-WT cell extracts using anti-CycE antibodies was used as a negative control (#).* p<0,0001 vs WT (Student-t test).

As mentioned above, our results indicate the presence of at least three CK2 phosphorylation sites in the KAP protein. A panel of mutants was generated and phosphorylation analyzed in ^32^P-labeled PCT3 transfected cells to map KAP phosphorylation sites within the PEST sequence. Site-directed mutagenesis was performed using the KAP wild-type sequence (KAP-WT). Individual phosphorylation site mutants (Fig. 6A) and ST65/66AA and ST86/87AA double-site mutants (Fig. 6B) were tested. S65A and T66A each reduced KAP phosphorylation by around 30%, while the ST65/66AA double mutant provoked 60% inhibition. Similarly, individual S86A and T87A mutations each reduced phosphorylation by 10%, while ST86/87AA mutations decreased by aproximately 30%. The T60A mutant reduced KAP phosphorylation by around 20%. These data indicated that two phosphorylation domains, Thr60 and Ser65/Thr66, placed in the most N-terminal PEST sequence, exhibited the strongest impact on KAP phosphorylation. To confirm this point, mutants including T60A and ST65/66AA mutations (KAP-DM) or residues T60A, ST65/66AA and ST86/87AA, resulting in the PEST-free sequence KAP-TM form, were tested. Phosphorylation of KAP-DM and KAP-TM was as low as 20% and 10%, respectively (Fig. 6C). The residual phosphorylation/radioactivity observed in KAP-TM could be attributable to other CK2 phosphorylation sites located outside the PEST region. Immunoprecipitation with anti-phospho-Thr antibodies of KAP-WT and KAP-TM in transfected PCT3 cell extracts confirmed that the Thr residues mutated in KAP-TM participate in KAP-WT phosphorylation (Fig. 6D).

### Cyclosporine A-induced KAP degradation is affected by PEST sequences integrity

**Figure 7:**
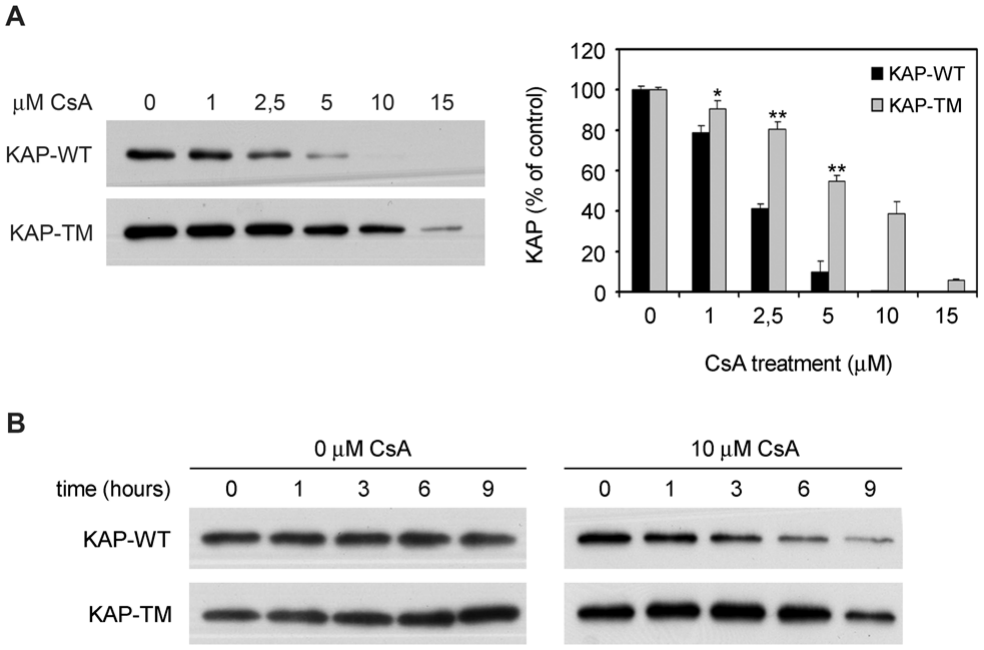
Cyclosporine A-induced KAP degradation is affected by PEST sequence integrity. (A) PCT3 cells transfected with the KAP-WT or the KAP-TM mutant vectors were treated with increasing doses of CsA (0, 1, 2.5, 5, 10 and 15 µM) for 20h. KAP protein levels in cell lysates were analyzed by western blot using anti-HA antibodies. PVDF membranes were stained with Coomassie Blue as a loading control (not shown). Normalized densitometric values of the western blot signals are shown in the right panel. Amount of KAP-WT and KAP-TM at time 0 h were taken as 100% in each case. Values at different times were referred to as a percentage of the total initial protein. * p<0,05 and ** p<0,001 vs dose-paired KAP-WT transfected cells (Student-t test).(B) Time-course study on KAP stability from a 1 to 9 h time period, using a single dose of CsA (10 µM) in PCT3 cells transfected with the KAP-WT or the KAP-TM mutant vectors. KAP protein was analyzed as in (A).

Next, we wondered whether CsA-induced KAP degradation would be influenced by KAP-phosphorylation. Dose-response CsA treatment showed KAP-TM to be was more resistant than KAP-WT (Fig. 7A). Time-course studies showed that in the absence of CsA, KAP-WT and KAP-TM levels were similar and remained stable for 9 h (Fig. 7B, left panel). By contrast, in the presence of a 10 µM CsA dose, KAP-WT decreased more rapidly than KAP-TM (Fig. 7B, right panel). These results indicated that CsA-mediated KAP degradation occurs in a dose- and time-dependent manner and that the effects were increased in the presence of intact PEST sequences in KAP.

### Calpain-mediated KAP degradation is favored by PEST sequences

**Figure 8:**
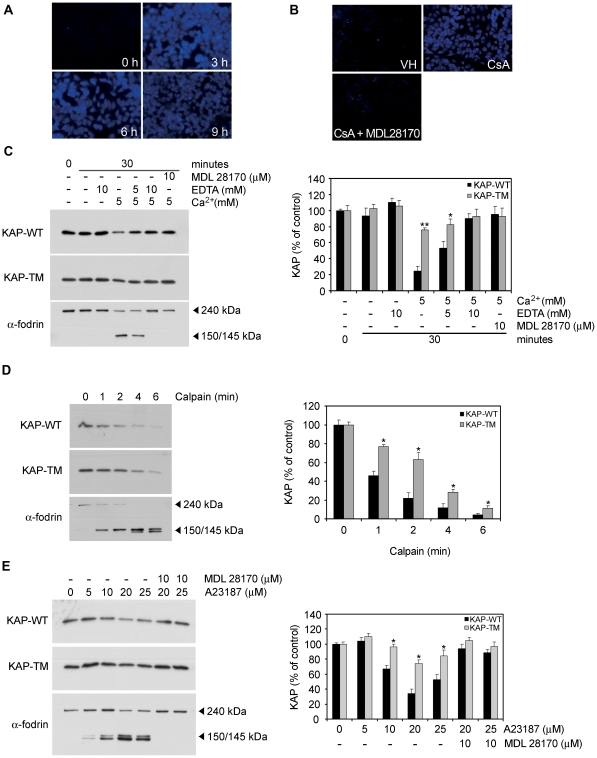
Calpain-mediated KAP degradation is favored by PEST sequences. (A) PCT3 cells were treated with 10 µM CsA for 3, 6 and 9 h and the fluorescence calpain substrate *t*-Boc-LM-CMAC (20 µM) was added for the last 45 min. Calpain activity was then analyzed by fluorescent microscopy. (B) PCT3 cells were treated with CsA 10 µM for 6 h alone or in presence of the calpain inhibitor MDL28170 (10 µM). Calpain activity was measured as above. (C) Transiently-transfected PCT3 cells with expression constructs containing the KAP-WT or the KAP-TM mutant were lysed and protein extracts prepared. Extracts were incubated in assay buffer containing 5 mM CaCl_2_, 5 or 10 mM EDTA and/or 10 μM of MDL 28170. KAP and α-fodrin proteins were detected by western blot using antibodies against the HA epitope or specific anti-α-fodrin antibodies. The right panel represents the signal quantifications, taking as 100% the densitometric values detected at time 0. * p<0,01 and ** p<0,001 vs treatment-paired KAP-WT transfected cells (Student-t test). (D) Transiently-transfected PCT3 cells with constructs expressing KAP-WT or KAP-TM were lysed 24h after transfection and crude extracts incubated with the purified m-calpain enzyme for different times. Steady-state protein levels of recombinant KAP-WT and KAP-TM or the endogenous α-fodrin were determined by Western blot assays. The activity of m-calpain was demonstrated by the efficient degradation of the α-fodrin protein upon exposure to the enzyme. Quantification assessed by densitometric analysis is represented in the right-hand panel of figure C. * p<0,01 vs time-paired KAP-WT transfected cells (Student-t test). (E) PCT3 cells transiently transfected with the KAP-WT and the KAP-TM constructs were exposed to increasing doses of ionophor A23187 and 5 mM CaCl2, in the absence or presence of the specific calpain inhibitor MDL 28170. Twenty-four hours post-transfection, and after 45 min of calpain activation treatment, cells were lysed and extracts analyzed by western blot assays to determine steady-state KAP levels. The 150/145 α-fodrin proteolyzed fragments appear under conditions that prompted activation of endogenous calpain in the cells. As above, densitometric analyses of western blots are represented in the lower panel of the figure. The situation control in each case was taken as 100% of protein expression levels and used as a reference for the remaining treatments. * p<0,01 vs dose-paired KAP-WT transfected cells (Student-t test).

As mentioned above, KAP phosphorylation sites for CK2 are inserted in PEST sequences which act as recognition signals for degradation by calpain. In an attempt to confirm calpain activation in response to CsA in PCT3 cells, a specific substrate that becomes fluorescent upon calpain-mediated degradation (t-Boc-LM-CMAC, Molecular Probes) was used in these cells. CsA treatment promoted calpain activation (Fig. 8A), which was specifically abrogated by the selective calpain inhibitor MDL28170 (Fig 8B).

In intact cells, calpain exists in cytosol as an inactive enzyme and translocates to membranes in response to increases in cellular Ca^2+^ level [24]. Our results show, that in PCT3 cell extracts, KAP-WT or KAP-TM were equally stable in the absence of Ca^2+^ (Fig. 8C, lanes 2 and 3). The presence of CaCl_2_ provoked strong decay in KAP-WT (Fig. 8C, lane 4) that was dose-dependently reversed by the concomitant presence of EDTA (Fig. 8C, lanes 5 and 6). Since effects were less prominent on KAP-TM, we hypothesized that disruption of PEST motifs partially protected KAP from degradation by an endogenous Ca^2+^-dependent protease. The pattern of α-fodrin degradation confirmed endogenous calpain activation (Fig. 3B). Moreover, the calpain selective inhibitor MDL 28170 prevented KAP and α-fodrin degradation in the presence of 5 mM CaCl_2_ (Fig. 8C, lane 7).

To further assess whether KAP-WT and KAP-TM had different calpain sensitivity, cell-free extracts from transiently-transfected PCT3 cells were incubated with purified recombinant m-calpain for different time periods (Fig. 8D). Under these conditions, KAP-WT and KAP-TM were also degraded by m-calpain, with KAP-TM being more resistant to proteolysis than KAP-WT (Fig. 8D). The different sensitivity of KAP-TM and KAP-WT to proteolysis was also assessed in intact cultured PCT3 cells treated with CaCl_2_ and increasing doses of ionophor A23187 in the absence or presence of MDL 28170. KAP-WT was degraded in the presence of ionophor A23187, in a dose-dependent manner (Fig. 8E, lanes 1 to 5), which was blocked by MDL 28170 (Fig. 8E, lanes 6 and 7). In these assays, KAP-TM was more resistant to degradation than KAP-WT. In summary, these findings shown in Figure 8 demonstrate that KAP is a target for calpain and that the mutation of PEST sequences significantly prevents KAP proteolysis mediated by this protease.

### KAP degradation by calpain in vitro is enhanced by CK2 phosphorylation

**Figure 9:**
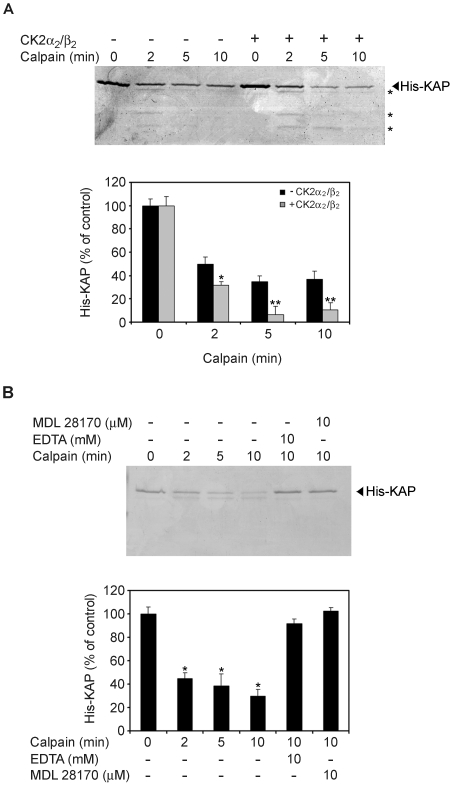
KAP degradation by calpain *in vitro* is enhanced by CK2 phosphorylation. (A) CK2 phosphorylation promotes *in vitro* degradation of KAP by calpain. 0.5 µg of mouse recombinant His-KAP-purified protein were incubated in the presence (lanes 5 to 8) or absence (lanes 1 to 4) of 2 pmols of CK2α_2_/β_2_ and ATP for 30 min. To each reaction, 0.18 U of m-calpain were added and incubated in assay buffer containing 5 mM CaCl_2_ for 0, 2, 5 and 10 min. Reactions were stopped by addition of loading buffer and products run in a 15% SDS-PAGE. Proteins were visualized by Coomassie Blue staining and graphic representation after densitometric analysis shown in the lower panel of the figure. It represents the normalized signal quantifications, taking as 100% the densitometric values detected at time 0. * p<0,05 and ** p<0,01 vs time-paired His-KAP incubated in the absence of CK2α_2_/β_2_ (Student-t test). (B) Non-phosphorylated KAP degradation is due to calpain. 0.5 µg of mouse recombinant His-KAP-purified protein were incubated with 0.18 U of m-calpain in assay buffer containing 5 mM CaCl_2,_ at different times; in these same conditions, at the 10 min time, 10 mM EDTA (lane 5) or 10 µM of MDL 28170 (lane 6) were also added. Degradation was visualized by Coomassie Blue in SDS-PAGE and quantification assessed by densitometric analysis (lower panel). * p<0,001 vs untreated His-KAP (Student-t test).

To determine whether KAP phosphorylation by CK2 would affect KAP degradation *in vitro*, and further demonstrate that phosphorylation rather than residue substitution in itself affected KAP degradation, *in vitro* assays using recombinant His-KAP protein and rat recombinant m-calpain were performed in the presence of 5 mM CaCl_2_. His-KAP was incubated with CK2 in phosphorylation buffer prior to the addition of calpain, and degradation compared with results obtained with non-phosphorylated His-KAP (Fig. 9A). Our results show that His-KAP incubated with CK2 was degraded more extensively than the non-phosphorylated one. In conclusion, these experiments show the recombinant KAP protein to be a good substrate for calpain and that phosphorylation by CK2 increases its degradation.

Recombinant His-KAP protein was incubated with m-calpain in the presence of EDTA or the specific calpain inhibitor MDL 28170 to further confirm that direct KAP degradation in the absence of CK2 phosphorylation was due to calpain (Fig. 9B). The presence of EDTA in the assay buffer completely blocked His-KAP degradation by m-calpain, thereby demonstrating absolute Ca^2+^ dependence for efficient KAP degradation. The specific calpain inhibitor MDL 28170 completely blocked substrate degradation, even in the presence of the protease (Fig. 9B).

### CK2 inhibitors prevent CsA toxicity in proximal tubule cells

**Figure 10:**
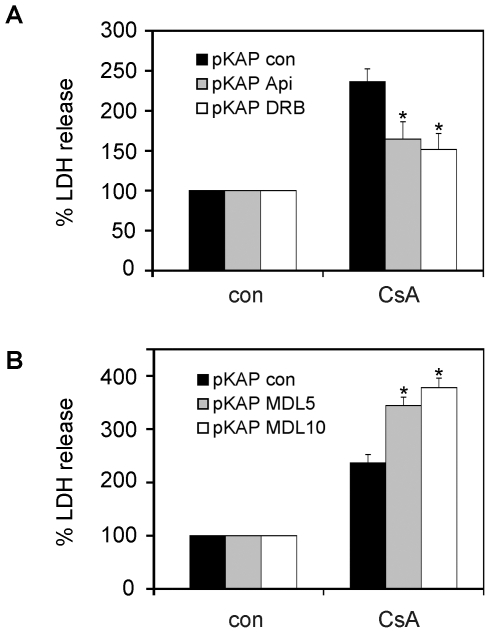
CK2 inhibitors prevent CsA toxicity in proximal tubule cells. The CK2 inhibitors apigenin and DRB (A) and the calpain inhibitor MDL 28170 (B) were used, at the pre-established non-toxic doses (50 µM for apigenin and DRB, and 5 or 10 µM for MDL 28170), on KAP transfected PCT3 cells to determine their effects on CsA-induced toxicity, measured by LDH release assays. The released LDH is expressed as the percentatge of total LDH measured after lysis of the cells. The data are then expressed taking the value of inhibitor or vehicle treated cells as 100% and are the mean ± S.E.M of three different experiments. * p<0,05 vs CsA-treated pKAP control transfected PCT3 cells (Student-t test).

Once demonstrated that: i) KAP diminishes CsA-induced toxicity, ii) KAP is phosphorylated by CK2 and iii) calpain-dependent degradation of KAP is favored by CK2 phosphorylation, we wondered what the effect of CK2 and calpain inhibitors would be on cell viability upon CsA treatment. MDL 28170 calpain inhibitor, apigenin and DRB CK2 inhibitors, at the pre-established non-toxic doses, were used on KAP transfected PCT3 cells to determine their effect on CsA-induced toxicity. As shown in Fig 10 (upper part), both CK2 inhibitors, apigenin and DRB, protect cells from toxicity. Unexpectedly, although MDL 28170 in itself is non-toxic, it became toxic in combination with CsA (lower panel Fig 10). Consequently, although this calpain inhibitor would preserve KAP degradation, its use would not be recommended owing to its toxic effects when combined with CsA.

### Discussion

Since CsA constitutes a very potent therapeutic tool, better understanding of the molecular mechanisms underlying undesirable kidney toxicity would be extremely important from a clinical point of view. While renal vasoconstriction is a characteristic of acute CsA toxicity and is largely reversible with dose reduction [25], an irreversible decline in kidney function associated with structural changes may also be observed after long-term CsA use [26]. Although the molecular mechanisms of CsA toxicity have not been completely elucidated, clinical and experimental studies have provided accumulated evidences for a direct effect of CsA on proximal tubule epithelial cells [27], [28] that has been associated with chronic renal failure [29]. Morphologic evidence suggests that early sublethal tubular damage is confined to the S3 segment of the proximal tubule [14]; however the reason for this specificity has not been unraveled. Results presented in this paper indicate that CsA-induced downregulation of KAP, which occurs specifically in the S3 segment, is associated with expression of kidney injury marker KIM-1. Moreover, threshold maintenance of KAP levels achieved in CsA-treated KAP Tg mice protects against the tubular injury observed in control littermates. KIM-1 is more sensitive and represents a much earlier kidney injury marker than SCr, BUN and urinary NAG in multiple rat models of kidney injury, thereby indicating that KIM-1 measurements may facilitate sensitive, specific and accurate prediction of nephrotoxicity in preclinical drug screening [18]. Significant KIM-1 overexpression in chronic CsA nephropathy characterized by tubular injury in rats has also been observed [30]. Our results suggest that KAP exerts a protective effect *in vivo*, and that KAP degradation by CsA might represent a novel mechanism that underlies CsA-induced toxicity in the S3 segment of the proximal tubule. Understanding KAP degradation mechanisms would be extremely useful for developing new therapeutic strategies to prevent CsA toxicity in the kidney while maintaining their immunosuppressive effects.

Previous analysis of the KAP primary sequence reported a putative PEST sequence encompassing 83–102 residues [3]. A more detailed analysis revealed a new PEST sequence between residues 53 and 89 with a pest score of 6.35. PEST sequences are rich in proline (P), glutamic acid (E), serine (S) and threonine (T) residues, are associated with proteins with a short half-life, and act as signals for degradation via proteosome or calpain. Some PEST sequences appear to be constitutive proteolytic signals, as for example, the carboxyl terminus of mouse ODC [22]. However, many PEST sequences are conditional signals, and can be activated in a number of ways [22]. Different molecular mechanisms, such as ligand binding [31], exposure to light and phosphorylation [22] have been described as activating this process. The paradigm of PEST-containing protein regulated by phosphorylation might be Ι| Bα. PEST-dependent I| Bα degradation involves CK2 phosphorylation of serine/threonine residues within the PEST domain of I| Bα that triggers calpain-mediated degradation [32]. Thus, we wondered whether KAP degradation occurred by a process similar to I| Bα. Our results show that calpain is activated by CsA both *in vitro* and *in vivo* and that calpain is involved in CsA-induced KAP cleavage. Calpains are intracellular cysteine proteases that play crucial roles in basic physiologic and pathologic processes [24]. Calpain has been reported to mediate ischemic/hypoxic injury in brain [33], kidney [34], liver [35] and myocardium [19]. Cytoprotection by calpain inhibitors has been observed during hypoxia in rat renal proximal tubules [36], cerebrocortical neurons [37] and rat hepatocytes subjected to anoxia. Calpain inhibitors have also been found to be effective in blocking cell death induced by a diverse group of toxicants in rat and rabbit proximal tubules [38]. Calpains are calcium–dependent proteases and, as for ischemic injury, levels of intracellular calcium have proved to play an important role in CsA toxicity [39], [40]. Reduction in extracellular calcium levels in the media or addition of calcium entry antagonists protected against CsA–induced cell damage [41]. Moreover, expression of calbindin–D28K, a cytosolic calcium binding protein, in cultured mouse proximal tubular cells protects against CsA toxicity, most likely through its buffering effects on intracellular calcium [42]. Since the critical substrates of calpains during cell injury/death remain unidentified, we postulate that KAP might be a potential target for understanding the molecular mechanisms underlying injury in proximal tubule cells.

The KAP PEST sequence presents residues Thr60, Thr66 and Thr87 as potential phosphorylation sites for CK2. Ser65 and Ser86 can also be considered potential CK2 sites according to the requirements fulfilled by other CK2 phosphorylated proteins [23]. CK2 is a ubiquitous, constitutively-active Ser/Thr protein kinase with a great diversity of substrates involved in a wide range of cellular functions [23], [43]. Several short-life proteins containing PEST sequences have been found phosphorylated by CK2 [44]–[46]. Our results show that CK2 phosphorylated KAP in cultured PCT3 cells and *in vitro* phosphorylation assays. Site-directed mutagenesis of residues located in KAP PEST sequences reduced KAP-TM mutant (T60A, ST65/66AA and ST86/87AA) phosphorylation levels to 10% of those found in KAP-WT. Moreover, mutation of these residues strongly diminished the PEST score of these sequences. We observed that KAP-TM was more resistant than KAP-WT in the following experimental conditions: i) cell-free extracts in the presence of calcium; ii) cell-free extracts incubated with purified recombinant m-calpain; iii) intact cultured cells incubated with calcium in the presence of the ionophore A23187; and iv) intact cells treated with CsA. Taken together, these results support the involvement of PEST sequences in KAP recognition by calpain. It is noteworthy that in all the cases mentioned above, KAP-TM was still partially degraded, which suggests that residues other than those mutated in the KAP-TM could contribute to calpain-mediated KAP degradation.

The implication of CK2 phosphorylation in KAP degradation was reinforced by the fact that His-KAP incubated with CK2 was degraded more extensively than the non-phosphorylated His-KAP. Since the latter was also degraded when exposed to calpain, our results suggest that although KAP degradation by calpain is enhanced by CK2 phosphorylation, this is not an absolute requirement. The role of PEST sequences underlying calpain substrate recognition is not fully understood. Mutations of PEST regions either abrogate substrate sensitivity to calpain [31], [47] or have no effect at all [48], [49]. Moreover, a significant fraction of calpain substrates have no PEST region at all [49]. Calpains are puzzling in that their requirement for calcium in vitro is considerably greater than most estimates of cytosolic calcium levels [49]. It was initially proposed that PEST regions could sequester calcium because of their negative charge and thereby present calpains not only with susceptible bonds but with the required co-factor as well [50]. In this respect, CK2 phosphorylation within PEST sequences could bring, through phosphate group addition to targeted residues, extra negative charges that could enhance calcium sequestration and thus foster calpain activation.

This work provides a novel mechanism for CsA-induced toxicity in proximal tubule cells based on KAP degradation by calpain, favored by CK2 phosphorylation of KAP PEST sequences. KAP-mediated protection against CsA-induced injury in proximal tubule cells, both in KAP Tg mice and in KAP transfected cells in culture, indicates that mechanisms either promoting KAP expression or preventing KAP degradation could represent specific therapeutic approaches to protecting proximal tubule cells from CsA damage and cardiovascular-related events. We previously reported that KAP Tg mice show hypertension and renal alterations including focal segmental glomerulosclerosis, proteinuria, glycosuria, and fibrosis [15]. While this phenotype appears at 6 to 8 months of age, likely due to sustained chronic KAP over-expression and increased oxidative stress exposure, the younger KAP Tg mice used in the present study did not show any sign of injury at 10–14 weeks of age, as observed by KIM-1 expression (Fig 1). The fact that sub-optimal KAP levels correlate with proximal tubule cell injury in CsA-treated mice or that supra-optimal KAP levels promote augmented oxidative stress and hypertension in Tg mice indicates the importance of maintaining KAP levels under tight regulated control. Besides the complex transcriptional regulatory mechanisms that control KAP mRNA levels in the kidney, we propose herein that KAP degradation by calpain after CK2 phosphorylation represents a novel layer of fine-tuning control on KAP protein levels and, therefore, a potent system to quickly respond to pathophysiologic demands. Furthermore, we propose that both KAP and CK2 could represent new therapeutic targets to ameliorate CsA toxicity in proximal tubule cells of the kidney.

## Materials and Methods

### Ethics statement

All studies were in compliance with the rules of the European Union and the US Department of Health and Human Services Guide for the Care and Use of Laboratory Animals. All procedures were approved by the authors' Institutional Review Board on Animal Health: Comitè Ètic de Experimentació Animal (CEEA) Hospital Universitari Vall d'Hebron. Permit Number FIS PI08/1351.

### DNA constructs

The pHA-KAP-HA mammalian expression vector contains the KAP cDNA. Site-directed mutagenesis was performed by PCR using the pHA-KAP-HA as template and overlapping primers encoding the mutations, following the manufacturer's instructions. Mutations were verified by DNA sequencing.

KAP was also subcloned into pET-14b (Novagen) with a His_6_ tag in the N-terminal domain (pET-14b/KAP). Human CK2α and CK2β subunits were cloned in pQE-30 vector 51]. KAP, CK2α and CK2β proteins were expressed in Escherichia coli and purified as described 52].

### Cell culture, transfection and siRNA experiments

PKSV-PCT (PCT3 clone) were cultured and transfected as described 7,8]. CK2α siRNA oligonucleotides were from Qiagen and silencing performed as indicated. CK2α evels were detected with anti-CK2α antibodies (Santa Cruz Biotechnologies).

### Phosphorylation assays in cultured cells

pHA-KAP-HA transfected cells were labeled *in vi*vo with [^32^P]orthophosphate (Amersham Biosciencies) in the presence, where indicated, of protein kinase CK2 inhibitors Apigenin and DRB (5,6-dichlorobenzimidazole riboside) (Sigma). Lysate supernatants, incubated with anti-HA high-affinity antibody (Roche Molecular Biochemicals), were precipitated with slurry protein G-Sepharose and proteins transferred to PVDF membranes. ^32^P-labeled KAP-HA was detected by autoradiography. Membranes were then incubated with anti-KAP antibodies and the blot was developed using chemiluminescence (ECL Plus, Amersham Pharmacia Biotech) and exposed to X-ray film (Kodak). Quantifications were made with a BioRad GS800 Calibrated Densitometer and ImageQuant software (Molecular Dynamics) and phosphorylation normalized by dividing the radioactivity incorporated (into KAP-HA) by the immunoreactive density of KAP-HA.

Alternatively, transfected cells were lysed in precipitation buffer and immunoprecipitated with anti-pThr antibody or anti-CycE antibody (negative control). Immunoblotting was performed with anti-KAP. 1 µg of total cell lysate protein was also loaded onto the gel and labeled as the INPUT.

### KAP stability after CsA treatments

Eighteen hours post-transfection, PCT3 cells expressing KAP-WT or KAP-TM were treated with increasing doses of CsA (Sandinmun, Roche) (0, 1, 2.5, 5, 10 and 15 µM) for 20 h. Protein levels in extracts were examined by Western blot with anti-HA antibody. KAP stability was also analyzed in transfected cells incubated with 10 µM CsA at different time points alone or in combination with either calpain or caspase inhibitors (MDL28170 or Z-VAD-FMK, respectively).

### *In vitro* degradation assay

Lysates from transfected cells were incubated with digestion buffer, with or without 5 mM CaCl_2_, in the presence or not of EDTA or MDL 28170. In another set of experiments, extracts were incubated with purified recombinant m-calpain in digestion buffer (containing CaCl_2_). Reactions stopped at various time points were resolved by Western blot using anti-HA or anti-α-fodrin antibodies. For *in vitro* KAP phosphorylation and degradation assays, recombinant His-KAP samples were incubated in kinase buffer in absence or presence of the CK2 holoenzyme [53]. His-KAP was digested with recombinant m-calpain in digestion buffer with CaCl_2_ alone or in the presence of EDTA or MDL 28170. Reactions were stopped by addition of SDS-PAGE loading buffer and electrophoresed . Proteins were visualized by gel Coomassie Brilliant Blue staining.

### Calpain activity assays

pHA-KAP-HA transfected cells were washed with PBS, treated with increasing doses of A23187 (Sigma) and 5mM CaCl_2_ in absence or presence of MDL 28170 and lysed with RIPA buffer. Protein extracts were analyzed by Western blot with anti-HA and anti-α-fodrin antibodies.

To assay the direct effect of CsA on calpain activation, PCT3 cells were plated at 50-80% confluence on glass coverslips for 24h and then treated with CsA 10 µM from 0 to 9h, in the presence of *t*-Boc-LM-CMAC (20 µM, Molecular Probes) for the last 45min. Samples were observed under a fluorescent microscope (excitation 329nm, emission 409nm). Calpain-catalyzed cleavage of Boc-LM-CMAC creates a fluorescent product. The fluorescence intensity of the samples correlates with calpain activity.

### Animal experimental protocol

Ten to 14-week-old male KAP-Tg mice and their littermates (15), were treated for 28 days with vehicle alone (*n* = 6) or CsA 50 mg/kg/day by subcutaneous injection (*n* = 6). Another group of animals received a low-salt diet (0.01% sodium, Harlan, Tekland) and, after one week, a daily subcutaneous injection of CsA 50 mg/kg/day (*n* = 6) or vehicle alone (*n* = 6) for 21 days.

Mice were euthanized and kidneys obtained from each animal for further analysis, in accordance with the requirements of the Spanish Government and the European Community (Real Decreto 1201/2005: B.O.E. no. 252. 21/10/2005).

### Functional studies

Serum was collected by cardiac puncture at the time of euthanasia. Serum creatinine (SCr) and serum blood urea nitrogen (BUN) were determined at the Clinical Biochemistry Service of the Veterinary Hospital of the Universitat Autònoma de Barcelona.

### Immunohistochemical analysis

Tissue sections were analyzed by indirect immunoperoxidase staining as previously described [15] with anti-KAP and anti-PCNA monoclonal antibodies (Santa Cruz Biotechnology). Goat anti-KIM1 polyclonal antibody (R&D Systems) was detected using the LSAB staining method (Dako LSAB+ System-HRP) following the manufacturer's protocol. The number of PCNA- and KIM-1-positive tubules in each section was determined by counting positively-stained cells or tubules in 10 randomly chosen fields (x 200 magnification) per slide. KAP levels in Tg mice and littermates, under control conditions and following different injuries, were quantified using the ImageJ software (ImageJ 1.44p, National Institute of Health, Bethesda, Maryland, USA) [54], [55]. Statistical analysis of the staining intensity was made using an ANOVA one-way test for each diet , followed by a least significant difference post-hoc test (STATGRAPHICS Plus statistical package, Statistical Graphics Corp.).

### Cell viability assay

CsA cytotoxicity was measured by LDH release assay using a commercial kit (LDH kit, Roche,

Mannheim, Germany) according to the manufacturer's instructions. The released LDH is expressed as the percentatge of total LDH measured after lysis of the cells.

### Western blot analysis

Kidneys were homogenized in lysis buffer (40 mM Tris-HCl, 4% CHAPS, 8 M Urea). Western blot analysis was performed with anti-KAP polyclonal antibody [15], anti-actin monoclonal antibody (Sigma) and anti-α-fodrin monoclonal antibody (Biomol International). The protein content of cellular extracts was quantified by the Bradford assay. Total cell extract protein was run on SDS-PAGE gels, transferred onto PVDF membranes and incubated with the corresponding antibodies. The membranes were developed with the enhanced chemiluminescence method (Pierce, Rockford, IL, USA).

### Statistical Analyses

Statistical analysis of staining intensity in kidney tissue sections was made using an ANOVA one-way test for each diet, followed by a Tukey post-hoc test. Results from figures 3, 4, 5, 6, 7, 8, 9, and 10 are expressed as mean ± SEM (standard error of the mean) of at least three independent experiments. Statistical analyses were performed with commercially-available software (Statgraphics Plus, Manugistics, Rockville, MD, USA).

## Supporting Information

Table S1 **Percent change of functional parameters in each group in respect to vehicle.**
(PDF)
