# Supplementary material for: KAP Degradation by Calpain Is Associated with CK2 Phosphorylation and Provides a Novel Mechanism for Cyclosporine A-Induced Proximal Tubule Injury
Source: PLoS One. 2011 Sep 28;6(9):e25746. doi: 10.1371/journal.pone.0025746 (PMC3182248; doi:10.1371/journal.pone.0025746)
Supplement: Table S1 — Percent change of functional parameters in each group in respect to vehicle. (PDF) [file pone.0025746.s001.pdf]

Table S1: Percent change of functional parameters in each group in respect to vehicle.

		BUN % change	Sc % change
Standard diet 28d treatment	Littermate-VH	100 ± 19,95	100 ± 12,36
	Littermate-CsA	105 ± 27,71	96,51 ± 10,33
	KAP Tg-VH	100 ± 12,77	100 ± 11,48
	KAP Tg-CsA	110,11 ± 48,38	104,51 ± 16,23
Low salt diet 21d treatment	Littermate-VH	100 ± 29,04	100 ± 2,95
	Littermate-CsA	103,79 ± 54,08	100,46 ± 8,66
	KAP Tg-VH	100 ± 24,82	100 ± 9,19
	KAP Tg-CsA	102, 40 ± 44,91	93,18 ± 17,72

Data are mean percent change ± SD of six animals. VH, vehicle; CsA, cyclosporine A; BUN, blood urea nitrogen; Scr, serum creatinine.
